# Validity of different copeptin assays in the differential diagnosis of the polyuria-polydipsia syndrome

**DOI:** 10.1038/s41598-021-89505-9

**Published:** 2021-05-12

**Authors:** Clara Odilia Sailer, Julie Refardt, Claudine Angela Blum, Ingeborg Schnyder, Jose Alberto Molina-Tijeras, Wiebke Fenske, Mirjam Christ-Crain

**Affiliations:** 1 Departments of Endocrinology, Diabetology and Metabolism, University Hospital Basel, Petersgraben 4, 4031 Basel, Switzerland; 2 University of Basel, Basel, Switzerland; 3 Medical University Clinic, Kantonsspital Aarau, Aarau, Switzerland; 4 Department of Endocrinology and Nephrology, University of Leipzig, Leipzig, Germany; 5 Leipzig University Medical Center, IFB Adiposity Diseases, Leipzig, Germany

**Keywords:** Pituitary diseases, Endocrinology

## Abstract

The aim of this study was to correlate three commercially available copeptin assays and their diagnostic accuracy in the differential diagnosis of the polyuria-polydipsia syndrome. Analyzed data include repeated copeptin measures of 8 healthy volunteers and 40 patients with polyuria-polydipsia syndrome undergoing osmotic stimulation and of 40 patients hospitalized with pneumonia. Copeptin was measured using the automated Brahms KRYPTOR, the manual Brahms LIA and the manual Cloud Clone ELISA assay. Primary outcome was the interrater correlation coefficient (ICC) and diagnostic accuracy in the polyuria-polydipsia syndrome of the three assays. In healthy volunteers, there was a moderate correlation for the KRYPTOR and LIA (ICC 0.74; 95% CI 0.07 to 0.91), and a poor correlation for the KRYPTOR and ELISA (ICC 0.07; 95% CI − 0.06 to 0.29), as for the LIA and ELISA (ICC 0.04; 95% CI − 0.04 to 0.17). The KRYPTOR had the highest diagnostic accuracy (98% (95% CI 83 to100)), comparable to the LIA (88% (95% CI 74 to 100)), while the ELISA had a poor diagnostic accuracy (55% (95% CI 34 to 68)) in the differential diagnosis of the polyuria-polydipsia syndrome. The KRYPTOR and LIA yield comparable copeptin concentrations and high diagnostic accuracy, while the ELISA correlates poorly with the other two assays and shows a poor diagnostic accuracy for polyuria-polydipsia patients. The current copeptin cut-off is valid for the KRYPTOR and LIA assay. Our results indicate that interpretation with other assays should be performed with caution and separate validation studies are required before their use in differentiating patients with polyuria-polydipsia syndrome.

**Trial registration:** NCT02647736 January 6, 2016/NCT01940614 September 12, 2013/NCT00973154 September 9, 2009.

## Introduction

Copeptin (also known as CT-proAVP) is the c-terminal portion of pre-pro-vasopressin, the precursor of the pituitary hormone vasopressin, which is the main regulator hormone of the sodium-water homeostasis^1–4^. In comparison to the instable and difficult to measure hormone vasopressin, copeptin is easily handled, is stable at room temperature and provides a reliable measure of the bioactive hormone^1^. As copeptin mirrors the physiological and pathological features of the pituitary hormone vasopressin, despite its different half-time, it is considered a true surrogate marker for vasopressin and has been increasingly used in clinical research and routine instead^5,6^. There are two main clinical implications of copeptin: first, as copeptin and vasopressin correlate closely over a wide range of plasma osmolality, copeptin can be used as a marker for vasopressin activity in the sodium-water homeostasis^5,6^. In this regard, copeptin has been investigated in the differential diagnosis of the polyuria-polydipsia syndrome^7–9^. It has been shown that copeptin is able to differentiate between patients with central diabetes insipidus, i.e., lack of vasopressin, and primary polydipsia following the hypertonic saline or arginine infusion test with a higher diagnostic accuracy than the long-time used indirect water deprivation test^8,9^. Second, copeptin is elevated in critically ill patients, such as pneumonia, stroke or myocardial infarction^10,11^ and has been shown to predict outcome of these diseases, with higher copeptin levels correlating with an unfavorable outcome^11,12^.

As for many other hormones and biomarkers, different types of immunoassays exist to measure copeptin. While some assays show a high inter-assay correlation^13^, others may yield quite different results^14^. To the best of our knowledge, there is no study investigating the inter-assay correlation of different copeptin assays. This is especially important with regards to the copeptin cut-off used in the differential diagnosis of the polyuria-polydipsia syndrome.

Therefore, the aim of this study was to compare three commercially available copeptin assays in different settings: first, over a wide range of serum osmolality in healthy volunteers, second, in the differential diagnosis of the polyuria-polydipsia syndrome, and third, in critically ill patients with community acquired pneumonia.

## Methods

### Patients and controls

For this analysis, data was combined from three different studies: first, healthy volunteers undergoing a test protocol of osmotic stimulation with hypertonic saline infusion^5^; second, patients with polyuria-polydipsia syndrome undergoing a hypertonic saline infusion as differential diagnosis between patients with central diabetes insipidus and primary polydipsia^8^; third, severely ill patients admitted to the hospital with community acquired pneumonia^15^. Full details of the studies rationales, designs and statistical analyses have been published elsewhere^5,8,15^. All three studies were registered on ClinicalTrials.gov (NCT02647736/NCT01940614/NCT00973154), conducted in accordance to the declaration of Helsinki and Good Clinical Practice Guidelines, and approved by the local ethical committees of all participating sites (Switzerland: Ethics Committee Northwest and Central Switzerland (EKNZ), Ethics Committee of Berne (KEK); Germany: the Ethical Committee of the University of Leipzig, Ethics Committee of the Medical Faculty of the Julius-Maximilians-University Würzburg; Brazil: Ethics Committees of the Federal University of Minas Gerais). Written informed consent was obtained from each participant after full explanation of the purpose and nature of all procedures used.

### Study procedure and expected copeptin concentrations in the original studies

#### Healthy volunteers

Participants were recruited at two tertiary care hospitals in Switzerland and Germany from September 2012 to July 2016^5^. Participants received hypertonic saline infusion (3% NaCl) while plasma sodium and copeptin were measured every 30 min until plasma sodium increased to > 147 mmol/l^5^. Expected copeptin concentrations cover the range of normal plasma copeptin, i.e., 1.23 pmol/l to 40 pmol/l.

#### Polyuria-polydipsia syndrome

Patients with polyuria-polydipsia syndrome, i.e., patients with complete central diabetes insipidus or primary polydipsia, were recruited at 11 tertiary medical centers in Switzerland, Germany, and Brazil from July 2013 to June 2017^8^. Patients underwent a test protocol with hypertonic saline infusion (3% NaCl). Blood for the analysis of copeptin was sampled at baseline and once plasma sodium was > 149 mmol/l. According to the results of the indirect classical water deprivation test, patients’ history and therapy response, patients were diagnosed by experienced and board certified endocrinologists. Expected copeptin concentrations cover the lower range of plasma copeptin, 1.23 pmol/l to 6 pmol/l, while stimulated plasma copeptin allows for the differential diagnosis of the polyuria-polydipsia syndrome.

#### Severely ill patients

Patients hospitalized with community acquired pneumonia were recruited in seven tertiary care hospitals in Switzerland from December 2009 to May 2014^15^. Community acquired pneumonia was defined by a new infiltrate on chest radiograph and the presence of at least one of the following acute respiratory signs and symptoms: cough, sputum production, dyspnea, core body temperature of 38.0 °C or higher, auscultatory findings of abnormal breathing sounds or rales, leucocyte count > 10,000 cells per μl or < 4000 cells per μl. Patients had blood sampling for copeptin concentrations on admission to the medical emergency department^15^. Expected copeptin concentrations cover the range of high plasma copeptin, i.e., above 40 pmol/l.

### Laboratory measurements and copeptin assays

In all three studies, blood for copeptin analysis was sampled in EDTA plasma tubes, immediately centrifuged at 4 °C for 10 min at 4000 rpm and stored at − 80 °C until analysis. Storage time for severely ill patients was longest (5–10 years), moderate in healthy volunteers (4–8 years) and shortest in patients with polyuria-polydipsia syndrome (3–7 years). Analysis for this study was performed for each assay in one batch with the same freeze–thaw cycles. Several “research use only” (RUO) and few CE marked (in vitro diagnostic, IVD) copeptin assays are commercially available. Copeptin was measured in duplicates with the Brahms Copeptin proAVP KRYPTOR assay (short KRYPTOR), the Brahms CT-proAVP LIA assay (short LIA) and the Cloud Clone CT-proAVP ELISA assay (short ELISA). The first two assays are CE marked while the Cloud Clone is an example of a RUO, which are mainly based on ELISA technology.

#### The KRYPTOR

The KRYPTOR is a CE marked automated immunofluorescent assay for the quantitative determination of copeptin. The measurement principle is based on Time-Resolved Amplified Cryptate Emission Technology (TRACE), which measures the signal that is emitted from an immunocomplex with a time delay. The fluorescent signal is emitted once the donor and acceptor antibodies form an immunocomplex with the antigen. The signal is proportional to the copeptin concentration.

According to the manufacturer’s instructions for use, the limit of detection was assessed as being 0.69 pmol/l, the intra-assay coefficient of variation as ranging from < 15% to < 4% for copeptin concentrations of 2 pmol/l to > 50 pmol/l and the inter-assay precision as ranging from < 18% to < 5% for copeptin concentrations from 2 pmol/l to > 50 pmol/l.

#### The LIA

The LIA is a CE marked immunoluminometric assay for quantitative determination of copeptin. Two antigen-specific antibodies bind copeptin at two different binding sites of which one is a labelled (chemiluminescent) tracer and the other is fixed to the inner walls of the reaction tubes (capture antibody; coated tube system). During incubation, both antibodies react with copeptin to form a sandwich complex. The tracer is quantified by measuring the luminescent signal and the signal intensity is proportional to the copeptin concentrations bound to the capture antibody.

According to the manufacturer’s instruction for use, the limit of detection was assessed as being 0.4 pmol/l, the intra-assay coefficient of variation as being < 5% for copeptin concentrations of > 2 pmol/l and the inter-assay coefficient of variation as being < 10% for copeptin concentrations > 2.5 pmol/l.

#### The ELISA

The ELISA is a “research use only” sandwich enzyme immunoassay for the in vitro quantitative measurement of copeptin. The microplate is pre-coated with an antibody specific to copeptin. During the incubation, the specific antibodies react with copeptin which leads to a color change. The color change is proportional to the copeptin concentrations and measured with a spectrophotometer at a wavelength of 450 nm. The copeptin concentration is determined by comparing the results to a standard curve.

According to the manufacturer’s manual, the limit of detection was assessed as being 1.4 pmol/l, the intra-assay coefficient of variation as being < 10% and the inter-assay coefficient of variation as being < 12% for the whole copeptin measuring range.

### Statistical analysis

The full analysis set included the data where copeptin was measured with all three assays (n = 150). Results are shown as median and interquartile range (IQR) and number (n) with percentage (%), appropriately. Three-group comparison for paired samples was done using the Friedman test. Two-group posthoc comparison was done using the Wilcoxon signed-rank test for paired samples with p-value adjustment according to Bonferroni.

Assay agreement was assessed using the intraclass correlation coefficient (ICC), a correlation analysis used to assess the consistency of two observers measuring the same quantity, herein copeptin concentrations measured with the KRYPTOR, the LIA and the ELISA. An ICC < 0.5 was considered a poor correlation, an ICC between 0.50 and 0.75 was considered a moderate correlation, an ICC between 0.75 and 0.90 was considered a good correlation and an ICC > 0.90 was considered an excellent correlation^16^. Graphical representation of how well the three copeptin assays correlate and mean copeptin difference between the assays including the lower and upper confidence limits was performed using the Bland–Altman Plot and the Bland–Altman Statistics.

Differential diagnosis of patients with polyuria-polydipsia syndrome was conducted using the area under the receiver operating characteristic curve (AUC) to assess the sensitivity and specificity of each copeptin assay. The “diagnostic gold standard” was used with the final diagnosis of the previous publication using the validated copeptin cut off of 4.9 pmol/l^8^.

Statistical and graphical analyses were performed using the statistic program R Statistical Software^17^. Hypothesis testing was two-sided and p-values < 0.05 were considered statistically significant.

## Results

### Patients’ characteristics

We included 8 healthy volunteers with a total of 30 plasma copeptin measurements (8 at baseline and 22 with rising plasma osmolality until serum sodium > 147 pmol/l), 40 patients with polyuria-polydipsia syndrome, 20 patients with primary polydipsia and 20 patients with complete central diabetes insipidus, with a total of 80 copeptin measurements (40 basal and 40 osmotically stimulated copeptin measurements), and 40 severely ill patients with community acquired pneumonia, with a total of 40 copeptin measurements (all on admission to the medical emergency department). Baseline characteristics of the three patient cohorts are displayed in Table 1. The median plasma copeptin concentrations for all three assays are displayed in Table 2.

**Table 1:** Baseline Characteristics of all three cohorts.

	Healthy volunteers	Polyuria-polydipsia patients	Severely ill patients
Number of patients	8	40	40
Age (years)	25.5 [23.8, 35.2]	40.0 [28.8, 49.2]	85.0 [79.5, 89.2]
Male sex, n (%)	4 (50)	20 (50)	29 (72)
BMI (kg/m^2^)	25.5 (8.4)	26.5 (7.1)	25.6 (4.9)
Systolic blood pressure (mmHg)	119 (12)	123 (16)	120 (25)
Diastolic blood pressure (mmHg)	75 (8)	76 (9)	64 (14)
Heart rate (bpm)	68 (7)	73 (13)	89 (23)

Continuous variables are expressed as median [Interquartile range], categorical variables as number and percentage.

*BMI* body mass index, *n* number, *bpm* beats per minute, *mmHg* millimeter mercury.

**Table 2:** Median copeptin concentrations of all three patients’ cohorts at any measured timepoint using the three different copeptin assays.

	KRYPTOR	LIA	ELISA	p-value
**Healthy volunteers (n = 30)**				
Plasma copeptin (pmol/l)	14.7 [8.8, 20.6]	10.1 [7.1, 14.0]	48.1 [35.4, 59.0]	< 0.001*
KRYPTOR vs LIA	14.7 [8.8, 20.6]	10.1 [7.1, 14.0]	–	< 0.001*
KRYPTOR vs ELISA	14.7 [8.8, 20.6]	–	48.1 [35.4, 59.0]	< 0.001*
LIA vs ELISA	–	10.1 [7.1, 14.0]	48.1 [35.4, 59.0]	< 0.001*
**Severely ill patients (n = 40)**				
Plasma copeptin (pmol/l)	74.3 [50.5, 135.1]	70.9 [47.8, 108.6]	80.1 [70.2, 125.0]	0.025*
KRYPTOR vs LIA	74.3 [50.5, 135.1]	70.9 [47.8, 108.6]	–	0.023*
KRYPTOR vs ELISA	74.3 [50.5, 135.1]	–	80.1 [70.2, 125.0]	1
LIA vs ELISA	–	70.9 [47.8, 108.6]	80.1 [70.2, 125.0]	0.71
**Polyuria-polydipsia patients (n = 80)**				
Plasma copeptin (pmol/l)	2.9 [1.6, 10.3]	2.8 [0.7, 9.7]	17.9 [11.3, 28.9]	< 0.001*
KRYPTOR vs LIA	2.9 [1.6, 10.3]	2.8 [0.7, 9.7]		0.70
KRYPTOR vs ELISA	2.9 [1.6, 10.3]		17.9 [11.3, 28.9]	< 0.001*
LIA vs ELISA		2.8 [0.7, 9.7]	17.9 [11.3, 28.9]	< 0.001*

Copeptin was measured in duplicates with the KRYPTOR, the LIA and the ELISA. Continuous variables are expressed as median [interquartile range]. Three-group comparison was done using the Friedman Test. Posthoc two group comparison was done using the paired Wilcoxon Sign Rank Test with Bonferroni p-value adjustment.

*Statistically significant.

### Different copeptin assays in healthy volunteers

#### Median plasma copeptin concentrations

There was a significant difference between the three assays (p-value < 0.001), with the LIA reporting the lowest and the ELISA reporting the highest copeptin concentrations (posthoc analysis: KRYPTOR vs LIA p-value < 0.001; KRYPTOR vs ELISA p-value < 0.001; LIA vs ELISA p-value < 0.001).

#### Intra-assay comparison

**Figure 1:**
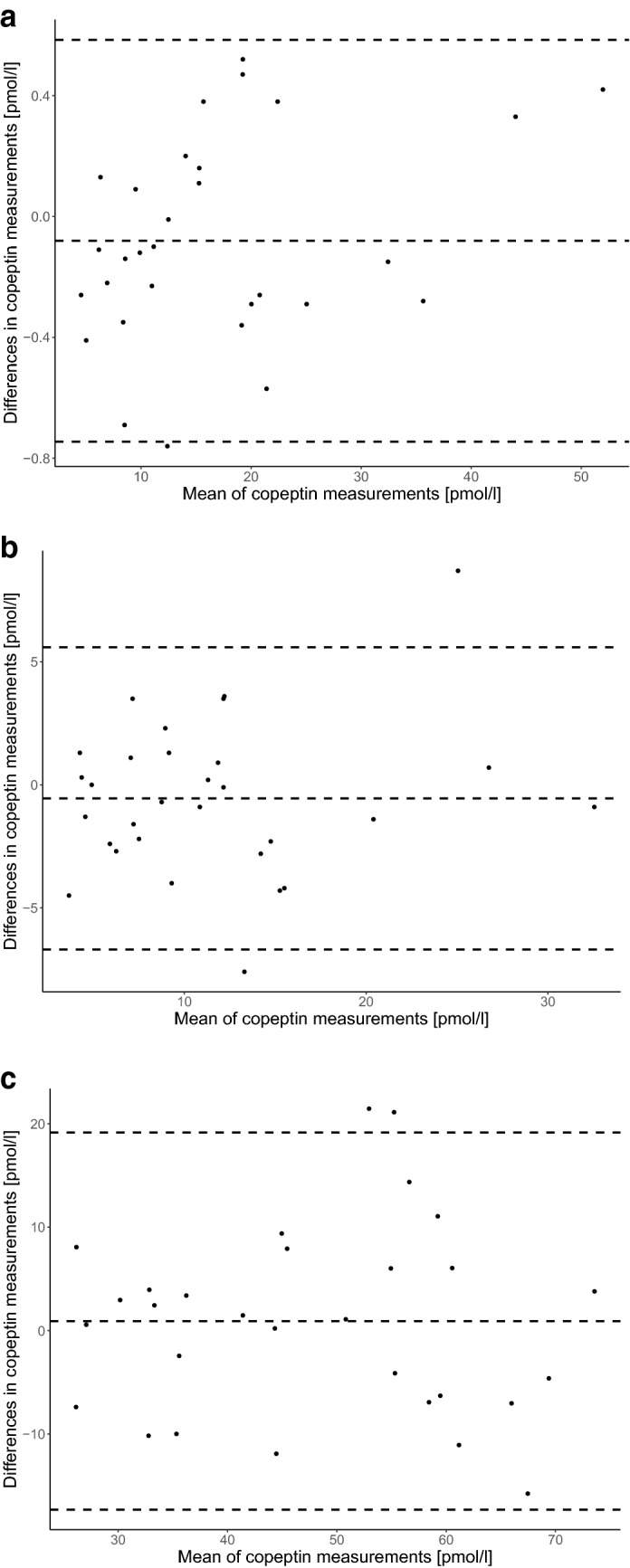
Intra-assay difference in copeptin concentrations in healthy volunteers. Bland–Altman graph of intra-assay difference in copeptin concentrations in healthy volunteers for (**a**) the KRYPTOR, (**b**) the LIA and (**c**) the ELISA. The dotted middle line represents the mean difference in copeptin concentrations between the two different assays (**a**): − 0.08 pmol/l (95% limits − 0.75 to 0.48), (**b**): − 0.55 pmol/l (95% limits − 6.69 to 5.59), (**c**): 0.92 pmol/l (95% limits − 17.32 to 19.16)). The outer dotted lines represent the 95% confident interval limits of agreement. (**a**) KRYPTOR 1 vs KRYPTOR 2, (**b**) LIA 1 vs LIA 2, (**c**) ELISA 1 vs ELISA 2.

There was an excellent correlation between the duplicated copeptin measurements using the KRYPTOR (ICC 0.99 (95% CI 0.99 to 1), mean copeptin difference − 0.08 pmol/l, (95% limits − 0.75 to 0.48)); an excellent correlation using the LIA (ICC 0.90 (95% CI 0.81 to 0.95), mean copeptin difference − 0.55 pmol/l (95% limits − 6.69 to 5.59)); and a good correlation using the ELISA (ICC 0.81 (95% CI 0.63 to 0.90), mean copeptin difference 0.92 pmol/l (95% limits − 17.32 to 19.16)) (Fig. 1a–c).

#### Inter-assay comparison

**Figure 2:**
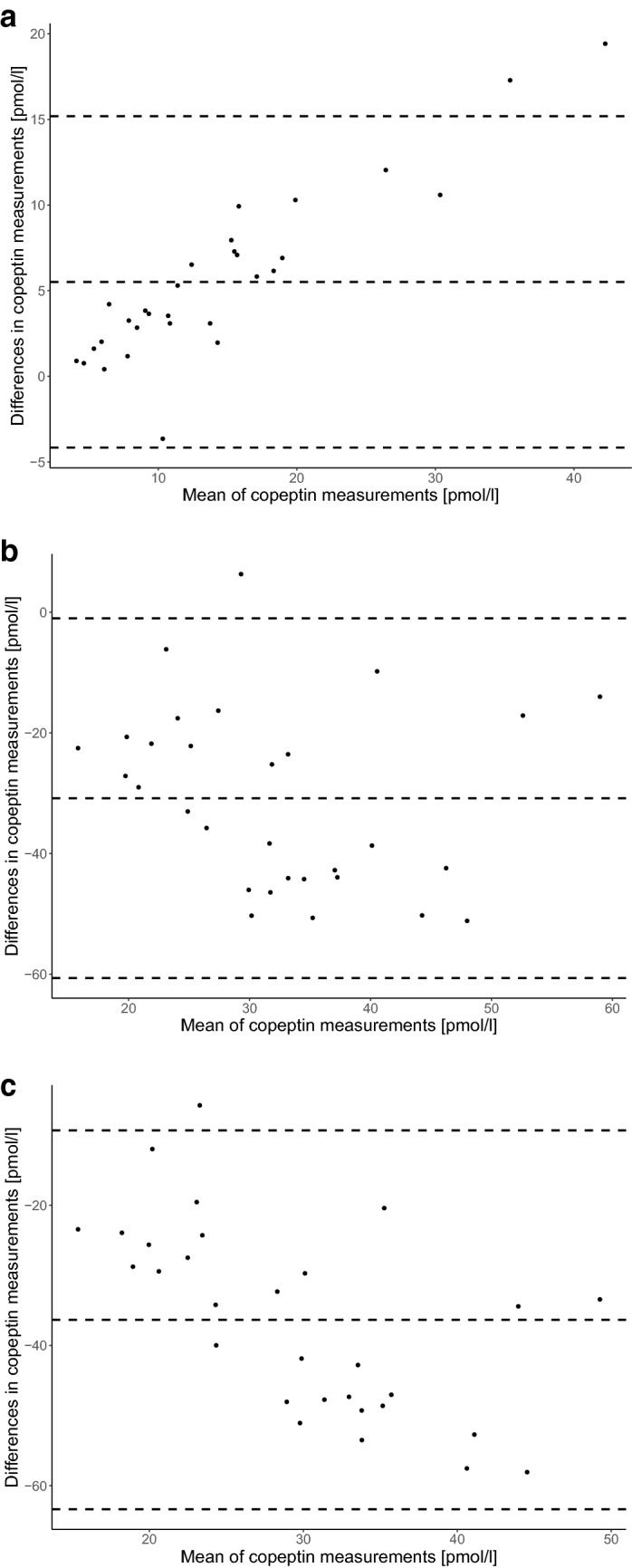
Inter-assay difference in copeptin concentrations in healthy volunteers. Bland–Altman graph of inter-assay difference in copeptin concentrations in healthy volunteers for (**a**) the KRYPTOR vs LIA, (**b**) the KRYPTOR vs ELISA and (**c**) the LIA vs ELISA. The dotted middle line represents the mean difference in copeptin concentrations between the two different assays ((**a**): 5.51 pmol/l (95% limits − 4.16 to 15.18), (**b**): − 30.8 pmol/l (95% limits − 60.63 to − 1.03), (**c**): − 36.34 pmol/l (95% limits − 63.37 to − 9.32)). The outer dotted lines represent the 95% confident interval limits of agreement. (**a**) KRYPTOR vs LIA, (**b**) KRYPTOR vs ELISA, (**c**) LIA vs ELISA.

There was a moderate correlation between the mean copeptin concentrations using the KRYPTOR and the LIA (ICC 0.74 (95% CI 0.07–0.91), mean copeptin difference 5.51 pmol/l (95% limits − 4.16 to 15.18)); a poor correlation using the KRYPTOR and the ELISA (ICC 0.07 (95% CI − 0.06 to 0.29), mean copeptin difference − 30.8 pmol/l (95% limits − 60.63 to − 1.03)); and a poor correlation using the LIA and the ELISA (ICC 0.04 (95% CI − 0.04 to 0.17), mean copeptin difference − 36.34 pmol/l (95% limits − 63.37 to − 9.32)) (Fig. 2a–c).

### Different copeptin assays in the differential diagnosis of polyuria-polydipsia syndrome

#### Median plasma copeptin concentrations

There was a significant difference between the three assays (p-value < 0.001). The posthoc analysis indicated that there was no significant difference between the KRYPOTR and the LIA (p-value = 0.70) but that there was a significant difference between the KRYPOTR and the ELISA (p-value < 0.001) as well as the LIA and the ELISA (p-value < 0.001).

#### Central diabetes insipidus

There was no significant difference in basal nor stimulated plasma copeptin concentrations in patients with central diabetes insipidus between the KRYPTOR or the LIA (basal p-value = 0.52, stimulated p-value = 1), but the ELISA reported significantly higher copeptin concentrations compared to both assays (basal and stimulated p-value < 0.001).

#### Primary polydipsia

There was no significant difference in basal plasma copeptin concentrations in patients with primary polydipsia between the KRYPTOR and the LIA (p-value = 1), but the ELISA reported significantly higher copeptin concentrations compared to the other two assays (p-value < 0.001). There was no significant difference between any of the assays for stimulated copeptin concentrations in patients with primary polydipsia (p-value = 0.1) (Table 3).

**Table 3:** Basal and stimulated copeptin concentrations of patients with central diabetes insipidus and primary polydipsia upon the hypertonic saline infusion test.

	KRYPTOR	LIA	ELISA	p-value
**Central diabetes insipidus (n = 20)**				
Basal plasma copeptin (pmol/l)	1.6 [1.4, 1.9]	0.8 [0.4, 2.0]	18.2 [8.8, 23.1]	< 0.001*
KRYPTOR vs LIA	1.6 [1.4, 1.9]	0.8 [0.4, 2.0]	–	0.52
KRYPTOR vs ELISA	1.6 [1.4, 1.9]	–	18.2 [8.8, 23.1]	< 0.001*
LIA vs ELISA	–	0.8 [0.4, 2.0]	18.2 [8.8, 23.1]	< 0.001*
Stimulated plasma copeptin (pmol/l)	1.7 [1.3, 2.6]	1.4 [0.4, 2.3]	17.2 [12.8, 28.6]	< 0.001*
KRYPTOR vs LIA	1.7 [1.3, 2.6]	1.4 [0.4, 2.3]	–	1
KRYPTOR vs ELISA	1.7 [1.3, 2.6]	–	17.2 [12.8, 28.6]	< 0.001*
LIA vs ELISA	–	1.4 [0.4, 2.3]	17.2 [12.8, 28.6]	< 0.001*
**Primary polydipsia (n = 20)**				
Basal plasma copeptin (pmol/l)	4.1 [2.6, 5.6]	3.4 [1.7, 5.9]	14.7 [11.1, 27.8]	< 0.001*
KRYPTOR vs LIA	4.1 [2.6, 5.6]	3.4 [1.7, 5.9]		1
KRYPTOR vs ELISA	4.1 [2.6, 5.6]		14.7 [11.1, 27.8]	< 0.001*
LIA vs ELISA		3.4 [1.7, 5.9]	14.7 [11.1, 27.8]	0.001*
Stimulated plasma copeptin (pmol/l)	20.4 [13.0, 27.8]	18.0 [11.5, 26.4]	23.0 [15.7, 34.6]	0.350
KRYPTOR vs LIA	20.4 [13.0, 27.8]	18.0 [11.5, 26.4]	–	1
KRYPTOR vs ELISA	20.4 [13.0, 27.8]	–	23.0 [15.7, 34.6]	0.34
LIA vs ELISA	–	18.0 [11.5, 26.4]	23.0 [15.7, 34.6]	0.34

Copeptin was measured in duplicates with the KRYPTOR, the LIA and the ELISA. Results are indicated as median [Interquartile range]. Three-group comparison was done using the Friedman Test. Posthoc two group comparison was done using the paired Wilcoxon Sign Rank Test with Bonferroni p-value adjustment.

*Statistically significant.

#### Diagnostic accuracy

The overall diagnostic accuracy of the hypertonic saline infusion test with the previously established copeptin cut-off of 4.9 pmol/l^7,8^ in the differential diagnosis of the polyuria-polydipsia syndrome was poor using the ELISA (55% (95% CI 34–68) (Table 4, Supplementary Fig. S1). The sensitivity (correctly identifying patients as having central diabetes insipidus) was 10% (95% CI 1, 32) and the specificity (correctly identifying patients as having primary polydipsia was 100% (95% CI 83, 100) using the ELISA. The diagnostic accuracy, sensitivity and specificity of the KRYPTOR and LIA are comparable to what has been shown in previous studies by Timper et al. and Fenske et al.^7,8^.

**Table 4:** Diagnostic performance of osmotically stimulated copeptin concentrations.

	Diagnostic accuracy		Sensitivity		Specificity		Positive predictive value		Negative predictive value	
	% [95% CI]	No./total no	% [95% CI]	No./total no	% [95% CI]	No./total no	% [95% CI]	No./total no	% [95% CI]	No./total no
KRYPTOR	98 [83, 100]	39/40	95 [75, 100]	19/20	100 [83, 100]	20/20	100 [82, 100]	19/19	95 [76, 100]	20/21
LIA	88 [74, 100]	35/40	80 [56, 94]	16/20	95 [75, 100]	19/20	94 [71, 100]	16/17	83 [61, 95]	19/23
ELISA	55 [34, 68]	22/40	10 [1, 32]	2/20	100 [83, 100]	20/20	100 [16, 100]	2/2	53 [36, 69]	20/38

A copeptin cut-off of 4.9 pmol/l was used for the differential diagnosis of central diabetes insipidus (≤ 4.9 pmol/l) and primary polydipsia (> 4.9 pmol/l). Copeptin was measured in duplicates using the KRYPTOR, the LIA and the ELISA. Diagnostic accuracy indicates correctly identifying patients as having central diabetes insipidus or primary polydipsia. Sensitivity indicates correctly identifying patients as having central diabetes insipidus. Specificity indicates correctly identifying patients as having primary polydipsia.

### Different copeptin assays in critically ill patients with community acquired pneumonia

#### Median plasma copeptin concentrations

There was a significant difference between the three assays (p-value = 0.025). The posthoc analysis indicated that there was a significant difference between the KRYPOTR and the LIA (p-value = 0.023) but no significant difference between the KRYPOTR and the ELISA (p-value = 1) nor the LIA and the ELISA (p-value = 0.71).

#### Intra-assay comparison

There was an excellent correlation between the duplicated copeptin measurements using the KRYPTOR (ICC 0.99 (95% CI 0.99 to 1), mean copeptin difference − 0.12 pmol/l (95% limits − 5.4 to 5.7)); an excellent correlation using the LIA (ICC 0.99 (95% CI 0.98 to 0.99), mean copeptin difference 3.43 pmol/l (95% limits − 14.42 to 21.80); and a good correlation using the ELISA (ICC 0.84 (95% CI 0.71 to 0.91), mean copeptin difference − 1.49 pmol/l (95% limits − 41.34 to 38.36)) (Supplementary Fig. S2a–c).

#### Inter-assay comparison

There was an excellent correlation between the mean copeptin measurements using the KRYPTOR and the LIA (ICC 0.94 (95% CI 0.89 to 0.97), mean copeptin difference 8.38 pmol/l (95% limits − 32.48 to 49.24)); a poor correlation between the KRYPTOR and the ELISA (ICC 0.44 (95% CI 0.16 to 0.66), mean copeptin difference 5.5 pmol/l (95% limits − 104.65 to 115.64)); and a poor correlation between the LIA and the ELISA (ICC 0.44 (95% CI 0.14 to 0.67), mean copeptin difference − 2.65 pmol/l (95% limits − 108.25 to 102.96)) (Supplementary Fig. S3a–c).

## Discussion

This study has four main findings: first, copeptin concentrations measured by the KRYPTOR and the LIA assays showed a good correlation over a wide range of copeptin concentrations. Second, copeptin concentrations measured with the ELISA only correlate poorly with both the KRYPTOR and the LIA, especially in the lower detection range of copeptin concentrations. Third, the KRYPTOR and the LIA measured copeptin concentrations have a high diagnostic accuracy in the differential diagnosis of the polyuria-polydipsia syndrome using the existing copeptin cut-off of 4.9 pmol/l. Fourth, the ELISA measured copeptin concentrations have a poor diagnostic accuracy in this differential diagnosis with an especially low sensitivity in correctly diagnosing patients with central diabetes insipidus.

The first copeptin assay was a chemiluminescence sandwich immunoassay with coated tubes, developed in 2006 by Brahms GmbH, Hennigsdorf/Berlin, Germany^1^ and validated using healthy volunteers and severely ill patients with sepsis^1^. Over the past years, additional copeptin assays have been developed such as the automated KRYPTOR^18,19^ and various ELISA^20–22^ tests. Consequently, there are now several commercial assays increasingly being used but a systematic correlation of these different assays has not yet been performed. Here, we compared three commercially available assays. First, a Copeptin proAVP KRYPTOR test, an assay that has been used, amongst others, in two recently published studies in the differential diagnosis of the polyuria-polydipsia syndrome^8,9,23,24^. This test is most often used in clinical studies. Second, a LIA, an assay that has been used, amongst others, in the differential diagnosis of the polyuria-polydipsia syndrome^7^ and as outcome predictor of stroke and sepsis^11,12,25^. This test was one of the first copeptin assays and used in early clinical studies on copeptin. Third, an ELISA, an assay that has been used in several research studies such as a predictor of treatment response in children with nocturnal enuresis, in children exposed to maltreatment and antihypertensive treatment response^22,26–28^. Several ELISA assays have been developed by companies for research use only. The chosen ELISA is one of the most commonly used copeptin ELISA assays.

Our results indicate that the KRYPTOR and the LIA have a high inter-assay agreement, which means that they yield comparable copeptin concentrations when measuring the same sample. Even though the KRYPTOR compared to the LIA measures on average higher copeptin concentrations, the difference is not clinically significant in the lower and mid-copeptin range, as evident by basal copeptin concentrations in patients with central diabetes insipidus and primary polydipsia. With higher copeptin concentrations, the discrepancy between the KRYPTOR and the LIA becomes more significant. The discrepancy in the lower range could be caused by a different minimal detection limit, for both cases, extreme hormonal concentrations in general are more difficult to measure^29–31^. An alternative explanation for the discrepancy between the KRYPOTR and the LIA might be that there is currently no international standardization to calibrate assays measuring copeptin concentrations, as it exists for other assays. Furthermore, different technologies (TRACE vs chemiluminescence) are used for the KRYPTOR and LIA. These different technologies may lead to different results with higher variability over the range of copeptin concentrations. Despite these discrepancies in copeptin concentrations between the two assays, their diagnostic accuracy in the polyuria-polydipsia syndrome was excellent and there was no significant difference in patients with central diabetes insipidus or primary polydipsia. The copeptin cut-off of 4.9 pmol/l was established in a study using the LIA (sensitivity of 94.0%, specificity of 94.4%)^7^ and validated in a recently published multicenter study using the KRYPTOR^8^ (sensitivity of 93.2%, specificity of 100%). In the latter study, a posthoc analysis indicated that a copeptin cut-off of 6.5 pmol/l had an even higher accuracy (sensitivity of 94.9%, specificity of 100%), supporting our finding that the KRYPOTR measures slightly higher copeptin concentrations but that both assays can be used interchangeably.

The ELISA on the other hand correlates poorly over the wide range of copeptin concentrations and specifically in the differential diagnosis of the polyuria-polydipsia syndrome. This is especially true for the lower detection range (copeptin concentrations < 10 pmol/l). In the differential diagnosis of the polyuria-polydipsia syndrome this leads to falsely diagnosing patients with central diabetes insipidus as having primary polydipsia. A misclassification in this case may lead to false treatment and a fatal outcome as fluid restriction, the primary treatment option for patients with primary polydipsia, is contraindicated in patients with central diabetes insipidus. When this assay was used to assess copeptin concentrations in other indications than the polyuria-polydipsia syndrome, the copeptin results were generally in the range between 10 and 20 pmol/l, indicating a higher range of copeptin concentrations. The higher copeptin concentrations also in other studies may indicate that the ELISA in general measures higher copeptin concentrations^22,26–28^. The higher results could be related to a higher rate of copeptin fragments or other substances that are measured with the ELISA, which has been described for other assays^14^ or the used antibodies detect other epitopes on copeptin than the KRYPTOR or the LIA.

Since the copeptin cut-off of 4.9 pmol/l for the polyuria-polydipsia syndrome was evaluated using the LIA and validated using the KRYPTOR, it is obvious that these assays have the highest diagnostic reliability. The ELISA as an easy-to-handle assay can be done in most laboratories, in contrast to the automated Copeptin proAVP KRYPTOR test which requires the KRYPTOR instrument. Importantly, however, based on our data, the copeptin cut-off of 4.9 pmol/l which is now integrated into different diagnostic algorithms of the polyuria-polydipsia syndrome^32^ cannot be used with the tested ELISA. Separate validation studies are required before this assay can be applied in the differential diagnosis of central diabetes insipidus.

A few limitations should be mentioned: we investigated three commercially available copeptin assays but cannot extrapolate our results to other copeptin assays. We aimed to use the most commonly used copeptin assays to best represent different measurement techniques. Second, we did not include a study where copeptin concentrations was used as outcome predictor. However, the results from severely ill patients indicate that in the higher copeptin range, correlation between the ELISA and the other two assays is better than at the lower end. This is also in line with findings from other immunoassay comparisons, where detecting higher hormone concentrations seems more reliable than lower hormone concentrations. Third, we only included patients with complete, but not partial central diabetes insipidus. The principal aim of this study was to compare three commercially available assays in the diagnosis of central diabetes insipidus. To get the best possible answer, we have, in a first step, included only the clear cases which are best discriminated, i.e., complete central diabetes insipidus and primary polydipsia.

In summary, the existing cut-off for the differential diagnosis of central diabetes insipidus following hypertonic saline infusion are validated for the LIA and the KRYPTOR measured copeptin concentrations. Other copeptin assays may not yield the same results and hence results must be interpreted with caution. Use of other copeptin assays requires separate validation studies.

## Supplementary Information

Supplementary Information.
