# Supplementary material for: Validity of different copeptin assays in the differential diagnosis of the polyuria-polydipsia syndrome
Source: Sci Rep. 2021 May 12;11:10104. doi: 10.1038/s41598-021-89505-9 (PMC8114908; doi:10.1038/s41598-021-89505-9)

SUPPLEMENTARY DATA

Validity of different copeptin assays in the differential diagnosis of the polyuria-polydipsia syndrome

[1,2]Clara Odilia Sailer, MD, PhD, [1,2]Julie Refardt, MD, [1,2,3]Claudine Angela Blum, MD, [1,2]Ingeborg Schnyder, MD, [1]Jose Alberto Molina-Tijeras, [4,5]Wiebke Fenske, MD and [1,2]Mirjam Christ-Crain, MD, PhD

[1]Departments of Endocrinology, Diabetology and Metabolism University Hospital Basel, Basel, Switzerland;

[2]University of Basel, Basel, Switzerland;

[3]Medical University Clinic, Kantonsspital Aarau, Switzerland;

[4]University of Leipzig, Department of Endocrinology and Nephrology, Leipzig, Germany;

[5]Leipzig University Medical Center, IFB AdiposityDiseases, Leipzig, Germany

Short title: Copeptin assay comparison

Corresponding author and reprint requests:

Prof. Mirjam Christ-Crain, MD, PhD

ORCID: 0000-0002-6336-0965

Department of Endocrinology, Diabetes and Metabolism, University Hospital Basel

Petersgraben 4, 4031 Basel, Switzerland

Phone: +41 61 265 50 78, e-mail: mirjam.christ-crain@usb.ch

Supplementary Figure 1: Receiver Operating Characteristic (ROC) Curves for the Hypertonic Saline Infusion Test

Shown are the ROC curves for the discriminative accuracy of the hypertonic saline infusion test for the three different assays. The green curve shows the discriminative accuracy for the KRYPTOR, the yellow curve shows the discriminative accuracy for the LIA and the red curve shows the discriminative accuracy for the ELISA. The gray diagonal lines represent the results that would be expected by chance alone.

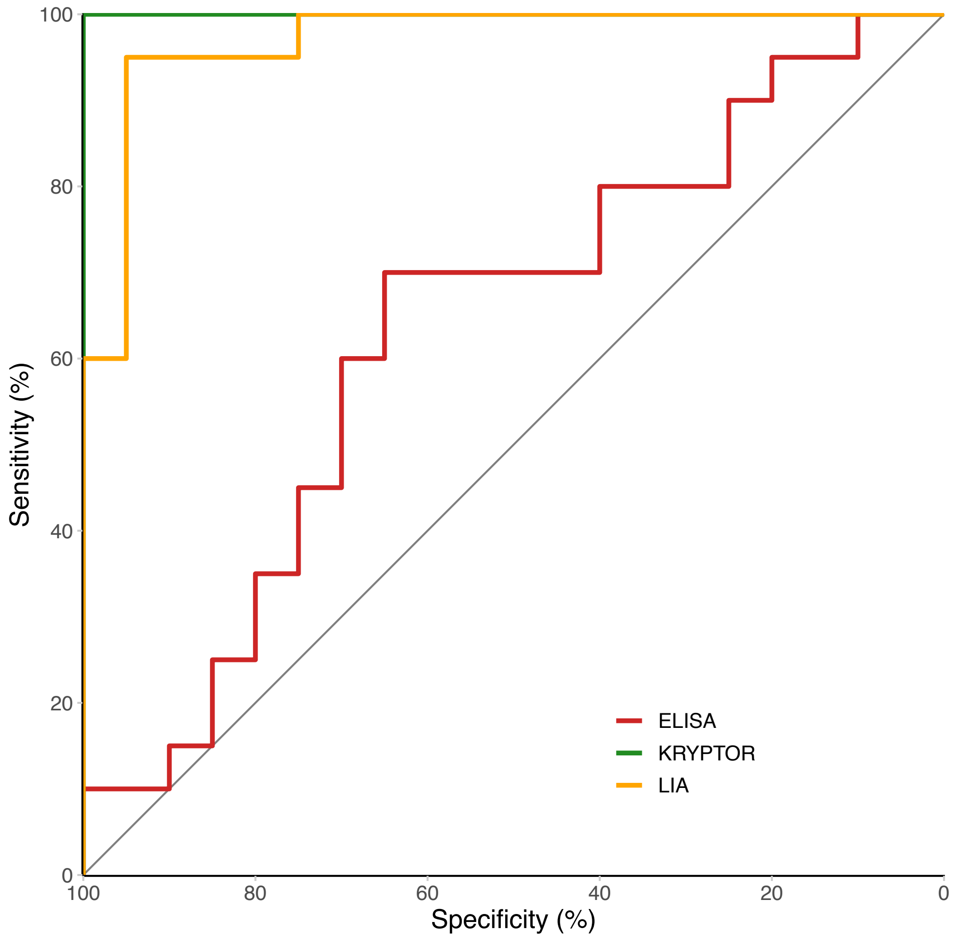

Supplementary Figure 2: Intra-assay difference in copeptin measurements for severely ill patients

Bland-Altman graph of intra-assay difference in copeptin measurements in severely ill patients for figure 2 A the KRYPTOR, figure 2 B the LIA and figure 2 C the ELISA. The dotted middle line represents the mean difference in copeptin measurements between the two different assays, the outer dotted lines represent the 95% confident interval limits of agreement (figure 2 A: mean difference -0.12 pmol/l (95% limits -5.4- 5.7), figure 2 B: 3.43 pmol/l (95% limits -14.42-21.80), figure 2 C: -1.49 pmol/l (95% limits -41.34-38.36)).

2 A – KRYPTOR 1 vs KRYPTOR 2

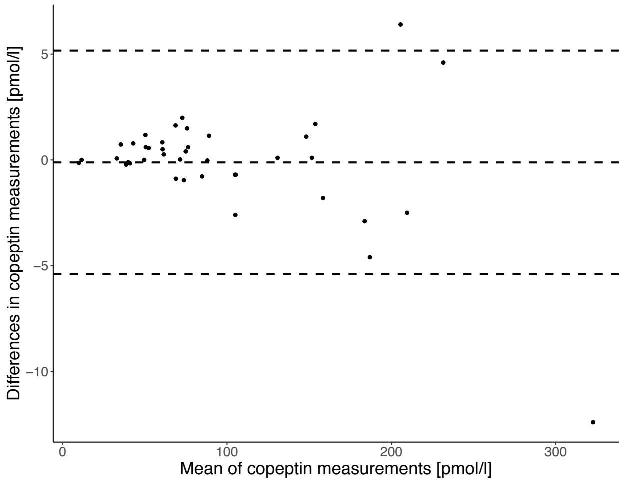

2 B – LIA 1 vs LIA 2

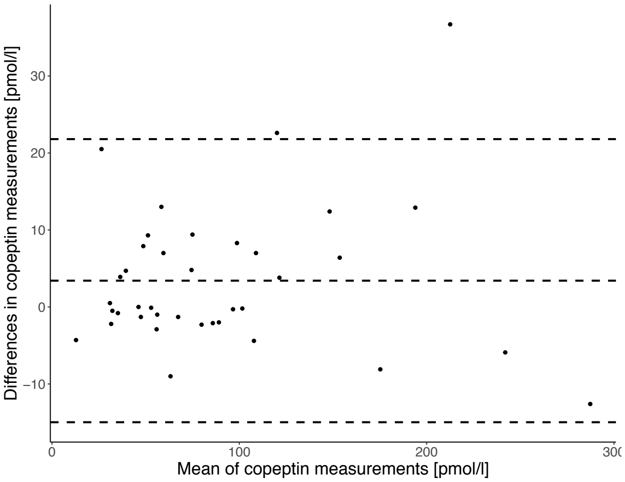

2 C – ELISA 1 vs ELISA 2

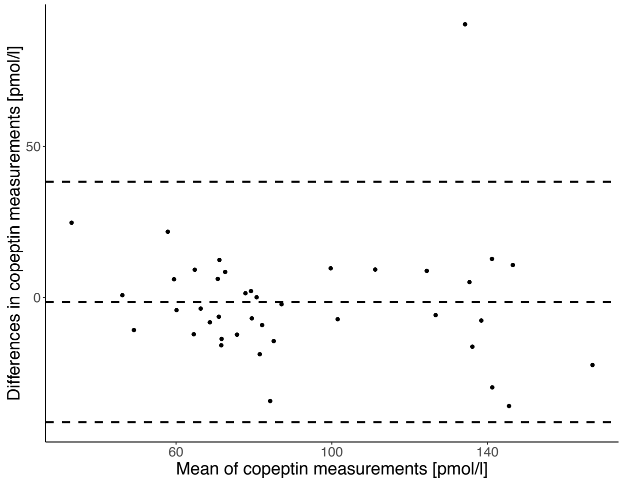

Supplementary Figure 3 – Inter-assay comparison in severely ill patients

Bland-Altman graph of inter-assay difference in copeptin measurements in severely ill patients for figure 3 A the KRYPTOR vs LIA, figure 3 B the KRYPTOR vs ELISA and figure 3 C the LIA vs ELISA. The dotted middle line represents the mean difference in copeptin measurements between the two different assays (figure 3 A: 8.38 pmol/l (95% limits -32.48-49.24), figure 3 B: 5.5 pmol/l (95% limits -104.65-115.64), figure 3 C: -2.65 pmol/l (95% limits -108.25-102.96)). The outer dotted lines represent the 95% confident interval limits of agreement.

3 A – KRYPTOR vs LIA

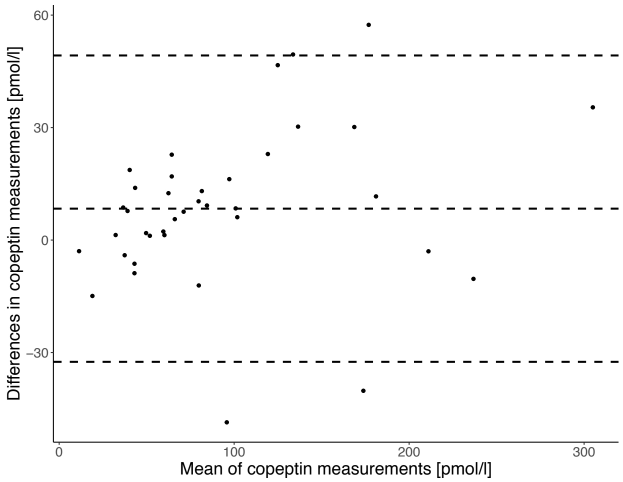

3 B – KRYPTOR vs ELISA

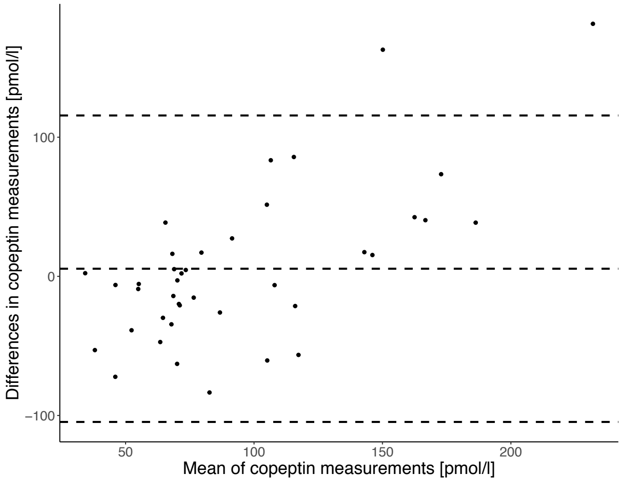

3 C – LIA vs ELISA

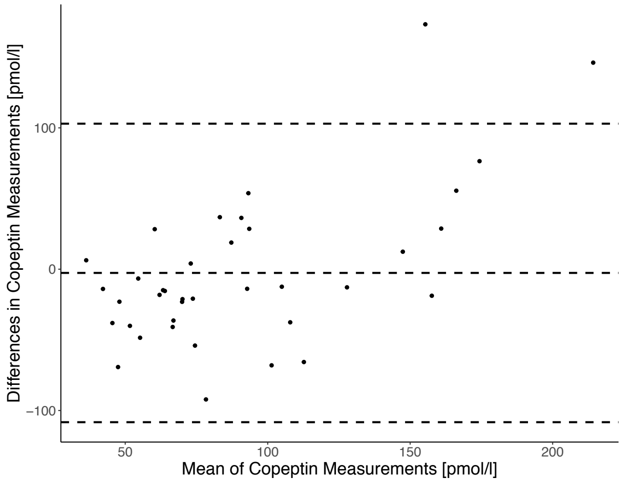

Supplement: Supplementary file 1 — Supplementary Information. [file 41598_2021_89505_MOESM1_ESM.pdf]
